# Winsome Brownie

**Published:** 1889-04

**Authors:** 


### WINSOME BROWNIE.

Dainty Brownie, winsome Brownie,
Brownie free and Brownie fair,
Be your pathway smooth and downy,
Everywhile and everywhere,
Wreathing still in soulful measure
And the melody of sound,
Words of passion, words of pleasure,
As a garland newly found
In love's consecrated ground,
By love's dainty fingers wound ;
Or a weird and wild cascade,
Arched above its misty glade
Into rainbows dreamy-bright,
By the sun's caressing light,
Thus alluring and beguiling
Out of sadness unto smiling,
Out of passion, out of wrong,
By your eloquence of song :—
Siren of the prairie wild,
Music's own devoted child,
Oh, my heart ! with love I crown ye,
Wooing, winsome, witching Brownie.

La Croix.

